# Low Birth Weight Impairs Acquisition of Spatial Memory Task in Pigs

**DOI:** 10.3389/fvets.2018.00142

**Published:** 2018-06-26

**Authors:** Sanne Roelofs, Ilse van Bommel, Stephanie Melis, Franz J. van der Staay, Rebecca E. Nordquist

**Affiliations:** ^1^Behavior and Welfare Group, Department of Farm Animal Health, Faculty of Veterinary Medicine, Utrecht University, Utrecht, Netherlands; ^2^Brain Center Rudolf Magnus, Utrecht University, Utrecht, Netherlands; ^3^Graduate School of Life Sciences, Utrecht University, Utrecht, Netherlands; ^4^Study Programme Applied Biology, HAS University of Applied Sciences, Den Bosch, Netherlands

**Keywords:** pigs, cognition, birth weight, sex differences, spatial learning, spatial holeboard task, cortisol

## Abstract

In commercial pig farming, an increasing number of low birth weight (LBW) piglets are born, due to selection for large litter sizes. While LBW piglets have a higher risk of pre-weaning mortality, a considerable number of these piglets survive to slaughter age. In humans, LBW is a risk factor for long-term cognitive impairments. In pigs, studies examining the post-weaning effects of LBW on cognition have reported contradictory results. Therefore, the current study aimed to assess the effects of LBW on cognitive development in pigs using an improved study design, by (1) testing a larger sample size than previous studies, (2) assessing acute and chronic stress responses to account for a potential altered stress response in LBW pigs, and (3) testing both female and male pigs to account for potential confounding effects of sex. Learning and memory of 20 LBW pigs and 20 normal birth weight (NBW) pigs, both groups consisting of 10 females and 10 males, were compared using a spatial holeboard task. In this task, pigs had to learn and remember the locations of hidden food rewards. After a pig had successfully acquired the task, it was presented with two successive reversal phases during which it was presented with a new configuration of reward locations. The holeboard allows for simultaneous assessment of working and reference memory, as well as measures of motivation, exploration, and behavioral flexibility. Mixed model ANOVAs revealed a transiently impaired reference memory performance of LBW pigs, implying they had more difficulty learning their reward configuration in the holeboard. Also, LBW piglets showed increased pre-weaning hair cortisol concentrations compared to their NBW siblings. No other effects of LBW were found. Sex had no direct or interaction effects on any measures of holeboard performance or stress. It is possible that the enriched housing conditions applied during our study had an ameliorating effect on our pigs' cognitive development. Overall, our results suggest LBW has a negative effect on post-weaning cognitive performance in pigs. This could have welfare consequences as cognitive skills are required for pigs to learn how to correctly respond to their environment.

## Introduction

Piglets born with low birth weight (LBW) are an increasingly common occurrence on commercial pig farms. This is a result of selection for increased sow fecundity, leading to larger litters. With increasing litter size, sows may be unable to provide sufficient nutrients and oxygen for the optimal development of all fetuses (1, 2). This explains the more frequent occurrence of LBW piglets in larger litters (3). Besides a sub-optimal prenatal development, LBW piglets also have a higher risk of pre-weaning mortality (4). While this results in a relatively higher number of LBW piglets dying during the farrowing stage compared to piglets with normal birth weight (NBW), there is still a considerable number of LBW piglets surviving to slaughter age at ~6 months old (5).

The sub-optimal development of LBW offspring has been associated with postnatal cognitive impairments in a variety of species. In humans, LBW has been linked to learning difficulties throughout adolescence (6, 7). Impaired cognitive development associated with LBW has also been studied in a variety of animal models, most frequently in rats and sheep [although contrary to pigs and humans, LBW has to be experimentally induced in these models—(8)]. For example, LBW has been linked to spatial memory deficits in rats (9). Together, these findings suggest that LBW could have a long-term impact on cognitive functioning. Pigs' prenatal brain development has similarities to humans', including a period of rapid brain growth *in utero* (10). It is possible that the cognitive impairments resulting from a sub-optimal intra-uterine environment are also comparable.

Understanding whether LBW also causes a long-term impairment of cognitive functioning in pigs is crucial, as such an impairment may influence their abilities to cope with housing and rearing conditions. Pigs are presented with multiple challenges to their learning and memory abilities in the common conditions of a commercial farm (11). For example, piglets have to learn how to acquire food from a feeder after being weaned (12), be able to recognize conspecifics and remember the organization of the dominance hierarchy to avoid unnecessary aggression (13, 14) and if available, be able to successfully interact with cognitive enrichment (15). Pigs need to be able to learn and remember how to interact with their environment, creating predictability, and controllability, which have been shown to reduce stress (16).

Studies examining the effects of LBW on cognitive development in pigs have produced contradictory results. A study comparing pre-weaning spatial learning abilities of LBW and NBW piglets found LBW to be associated with the expected impaired performance (17). After weaning, one study found LBW to be associated with the predicted cognitive impairments, with LBW pigs showing impaired reversal learning in a spatial learning task compared to NBW pigs (18). Other studies reported a comparable performance of LBW and NBW pigs, finding no effects of LBW on spatial learning ability (19) or associative learning (20, 21). There has also been a report of improved cognitive performance in LBW pigs, with LBW being associated with improved spatial learning (22). Together, these studies do not provide a consensus on the long-term impact of LBW on the cognitive development of pigs. Rather, they show the need for further replication of cognitive studies with LBW pigs, applying methodological improvements to increase the quality of results.

None of the studies assessing the long-term effects of LBW on cognitive development took a potential effect of their study subjects' sex into account. Although it is unlikely that sex in itself has an influence on baseline cognitive performance in pigs (23), it is possible that females and males perform differently under the influence of stress. For example, spatial learning and memory is typically impaired as a consequence of chronic stress (24). Such negative effects of chronic stress appear to be more prominent for males than females (25). The opposite has been found for acute stress, which causes a more detrimental effect on females' cognitive performance (26). Possible sex-dependent effects of stress are relevant when assessing cognition in LBW animals, as LBW may lead to altered functioning of the HPA-axis. For example, LBW piglets show increased plasma cortisol concentrations throughout the first week after birth (27, 28), and show an exaggerated cortisol response to a physiological stressor (administration of insulin or ACTH) at 3 months of age (29). Together, these results suggest that stressors may have a more detrimental effect on pigs with LBW. Considering that females and males may be differentially affected by such stressors, taking both sex of the study subjects and measures of stress into account is of importance when assessing the effects of LBW on cognition.

A suitable task to assess the effects of LBW on learning and memory in pigs is the spatial holeboard task. The holeboard is a free-choice maze task consisting of an open arena in which pigs have to learn and remember the locations of hidden food rewards (30). Such a task is highly ecologically relevant for pigs, as it is based on their natural foraging behavior (31). Furthermore, the spatial holeboard allows for simultaneous assessment of multiple behavioral variables. The most important cognitive measures provided by the spatial holeboard are reference and working memory. Reference memory is required for information that remains relevant over a longer time period, such as how well a pig remembers the locations of rewards and how many locations contain a reward (32). Reference memory can be quantified as the ratio between visits to rewarded and unrewarded locations (30). Working memory is required for information that is relevant for a shorter time span, such as which locations have already been visited within a single training trial (33). This information is irrelevant in subsequent trials and consequently, working memory must be reset between trials. Working memory can be quantified as the ratio between first visits and all visits (including revisits) to a location (30). Besides measures of spatial learning and memory, the holeboard can also be used to assess motivation (by measuring latency to first visit or the time interval between visits), exploration (by measuring which locations are visited) and behavioral flexibility [by applying a reversal of the task—(30)]. The spatial holeboard task has already successfully been applied to assess spatial cognition in pigs, showing it is sensitive enough to detect even mild cognitive impairments [e.g., (34)].

The current study aimed to assess the long-term effects of LBW on learning and memory in pigs, as assessed by the spatial holeboard task. Several improvements to previous studies were applied. First, a larger sample size was included, with 20 LBW and 20 NBW pigs being tested. This doubles the sample size used in previous studies to assess baseline effects of birth weight on post-weaning cognition (18, 19, 22). Second, as LBW pigs may suffer from an altered stress response, hair and salivary cortisol concentrations were included as measures of chronic and acute stress, respectively. Finally, female and male pigs were tested to account for a potential confounding effect of sex. Based on studies assessing cognitive effects of LBW in humans (6, 7) and earlier studies with pigs at various ages (17, 18) it was expected that LBW would cause an impaired cognitive development in pigs. This would result in decreased performance in the spatial holeboard, compared to NBW pigs. Furthermore, LBW pigs were expected to show an altered stress response, resulting in higher basal hair cortisol concentrations compared to NBW pigs and an exaggerated salivary cortisol increase after a stressor.

## Materials and methods

### Ethical note

All methods that demanded the handling of live animals were reviewed and approved by the local animal welfare body (Animal Welfare Body Utrecht) and were conducted in accordance with the recommendations of the EU directive 2010/63/EU.

### Animals

Twenty pairs of piglets [(Yorkshire × Dutch Landrace) × Duroc] from 15 different litters were selected from the commercial pig breeding farm of Utrecht University, resulting in 20 LBW pigs and 20 NBW pigs (10 pairs of females and 10 pairs of males). Selection occurred in two separate rounds of 20 piglets (ten LBW-NBW pairs) to ensure availability of LBW piglets. During each selection round, all piglets born over a period of 1 week were weighed within 24 h after birth. LBW piglets were selected based on three criteria: (1) a minimum of 1 SD below the average birth weight of the litter, (2) a minimum of 1 SD below the average birth weight of the study population, yielding a maximum birth weight of 1,050 grams, and (3) from a minimum litter size of 10 piglets. For each LBW piglet, a NBW sibling was selected based on two criteria: (1) of the same sex as the selected LBW piglet, and (2) a birth weight closest to the litter average. To increase food intake and thereby survival rates of LBW piglets, cross-fostering of non-selected siblings was applied when litter size exceeded the sow's number of functional teats. Additionally, all litters were provided with milk replacer at 2–3 days of age. One female LBW piglet and one male NBW piglet died of natural causes during the early stages of training in the holeboard. Their data was excluded from analysis, resulting in a final sample size of 38 pigs. Of these, one female LBW piglet could not participate in the second reversal phase due to lameness.

### Housing

The selected pigs were weaned and moved to the research facility (located next to the commercial farm), at ~4 weeks of age. They were housed in four adjacent pens (~4 × 5 m) in a naturally ventilated building. For each selection round, LBW and NBW pigs were housed separately. Pens had concrete floors and contained a covered piglet nest. Each day, the pens were cleaned and supplied with fresh straw bedding. To protect the piglets from the cold, the nest was equipped with rubber mats on the floor and transparent polyvinyl chloride (PVC) slats hanging in front of the entrance. Additionally, piglet nests contained heat lamps until the pigs were ~8 weeks old. Minimum and maximum temperatures were recorded daily outside the piglet nest and ranged from 0 to 27°C. To avoid effects of heat stress, pigs were only tested if they voluntarily entered the holeboard apparatus. Pigs received ^1^/_3_ of their daily food ration in the morning (prior to testing) and the remaining ^2^/_3_ in the afternoon (after testing). Water was provided *ad libitum*. Each pig had a number sprayed on its back to facilitate individual recognition of the pigs.

### Spatial holeboard task

#### Apparatus

**Figure 1:**
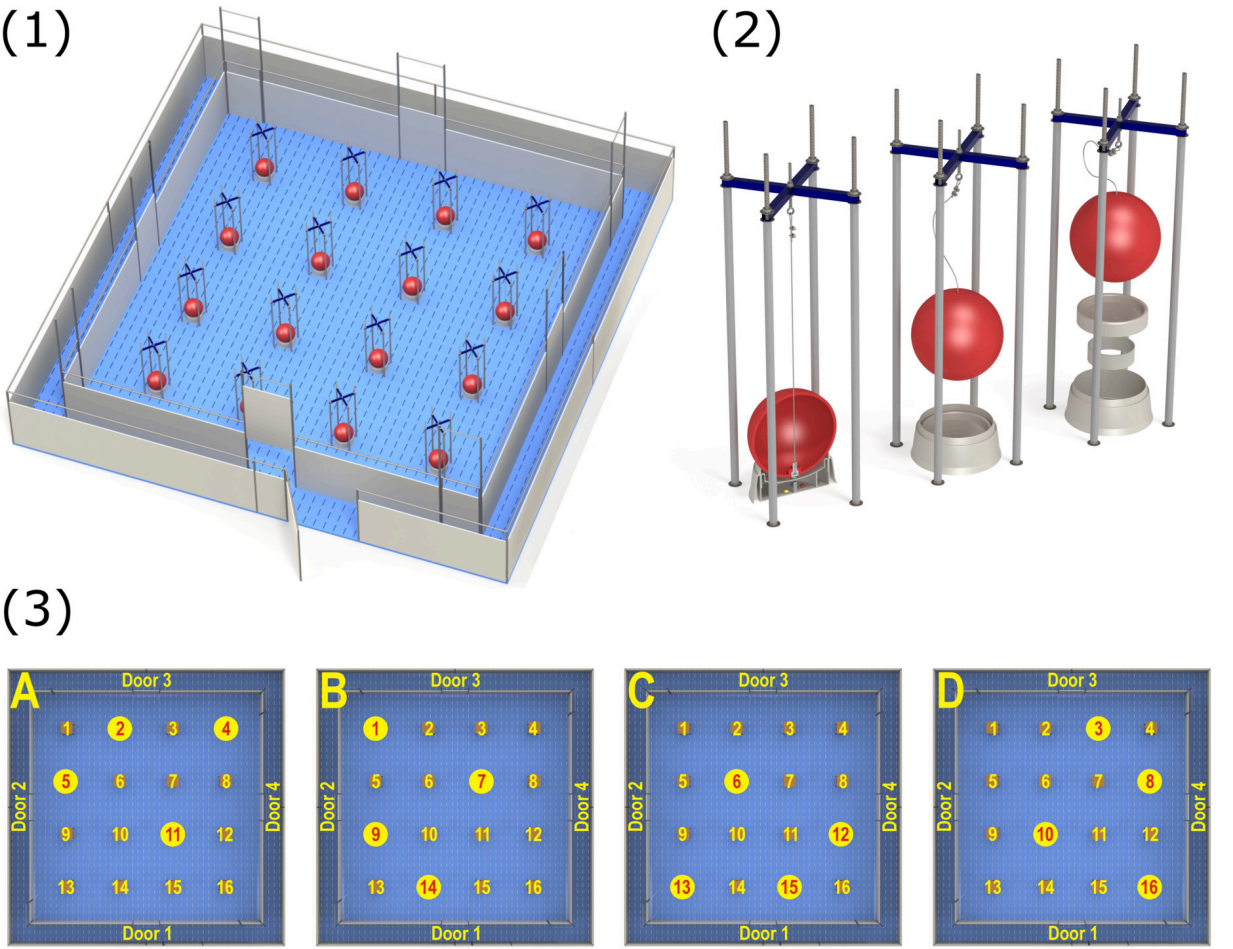
**(1)** Overview of the spatial holeboard apparatus. **(2)** Details of the food bowls (holes). Each bowl is covered with a red ball to hide visual cues. The bowls are equipped with a false bottom covering four candies to mask odor cues. **(3)** The different reward configurations (A–D) used during holeboard training. Baited holes are highlighted (illustrations: Yorrit van der Staay).

The holeboard apparatus (manufactured by Ossendrijver B.V., Achterveld, The Netherlands) consisted of a square arena (5.3 × 5.3 m) with a synthetic slatted floor, surrounded by synthetic walls (80 cm high). The holes in the arena consisted of 16 food bowls placed in a 4 × 4 matrix (Figure 1), in which food rewards could be hidden. Pigs could enter the arena to search for these rewards via a surrounding corridor (40 cm wide), which gave access to one of four guillotine doors (operated by a rope and pulley system) placed in the walls surrounding the arena. By using four different starting positions, pigs cannot rely on a fixed search pattern to solve the task (30). Instead, pigs had to rely on extra-maze cues (such as the position of the experimenter outside the arena) to orient themselves inside the holeboard. A baited hole would contain two chocolate candies (M&M's® Milk Chocolate) as a reward. Each food bowl was fixed with a false bottom, beneath which four candies were placed to avoid providing the pigs with scent cues about the locations of the baited holes (Figure 1). Additionally, each bowl was covered with a synthetic red ball (JollyBall Dog Toy, ø 24 cm, 1,400 g, Jolly Pets, Ohio, USA) to avoid visual discrimination between baited and non-baited holes. The pigs were trained to lift the ball off a food bowl in order to obtain the food reward. If a pig soiled the holeboard during testing, it was rinsed immediately to avoid the development of scent cues. Additionally, the entire holeboard was rinsed daily. During testing, visits to holes were automatically recorded using custom made software (SeaState5, Delft, The Netherlands). When a ball was lifted off a food bowl, the connection between a magnet in the ball and a sensor in the bowl was interrupted. This signal was registered by an interface (LabJack) and sent to a laptop. A revisit to a hole was only recorded if a pig visited another hole in between or if 10 s passed in between successive visits to the same hole.

#### Training and testing

After the pigs were moved to the research facility, training started by habituating the pigs to the presence of and being handled by the researchers. The pigs were then gradually habituated to being inside the holeboard apparatus. Initially, pigs were allowed to explore the holeboard in groups of ten. Group size was then gradually decreased until the pigs explored the holeboard individually.

Testing trials started when all pigs were able to lift the balls off the food bowls (at this point, pigs were ~8 weeks old). Each pig performed two consecutive trials daily. At the start of a trial, a pig was let into the corridor surrounding the holeboard arena. When it reached an open entrance into the arena (one of four entrances was randomly chosen prior to each trial), it could freely search for food rewards by visiting holes (i.e., lifting the ball covering a food bowl). A trial ended when a pig managed to find all rewards or when a maximum trial duration of 7.5 min had passed, whichever occurred first.

The holeboard experiment consisted of four consecutive phases: habituation (four trials), acquisition (44–76 trials), first reversal (24–44 trials) and second reversal (20 trials). During habituation trials, all 16 holes contained a reward. This encouraged the pigs to visit as many holes as possible during each habituation trial. After the habituation trials, the acquisition phase started. Each pig was assigned one of four possible reward configurations (Figure 1). A reward configuration consisted of a subset of four baited holes (the remaining 12 holes did not contain a food reward). Each pig continued training on its assigned reward configuration for the duration of the acquisition phase. Reward configurations were randomly assigned but balanced for birth weight category and sex.

There were two criteria for a pig to complete the acquisition phase, based on previous holeboard studies with pigs (18, 23): a pig had to (1) complete a minimum of 44 acquisition trials, and (2) reach a reference memory score of at least 0.7 (see section Ameliorating Effects of Enrichment, Variables for calculation of reference memory) for two consecutive training days (consisting of four consecutive trials). This criterion performance indicated a pig had successfully learned the locations of the four baited holes. After completing the acquisition phase, a pig was assigned a new reward configuration for the first reversal phase (Table 1). The pigs now had to learn to retrieve their rewards in a new set of holes (e.g., a pig that was trained on configuration A during acquisition was now switched to configuration C). Pigs could complete the first reversal phase after a minimum of 24 reversal trials and reaching the same criterion level for reference memory performance that was set during the acquisition phase. After completing the first reversal phase, a second reversal was applied where pigs were again trained on a new reward configuration (Table 1).

**Table 1:** Combinations of reward configurations used for the holeboard experiment.

**Combination**	**Phase**		
	**Acquisition**	**1st reversal**	**2nd reversal**
1	A	C	B
2	B	D	C
3	C	A	D
4	D	B	A

#### Behavioral variables

For each trial in the holeboard, the following variables were analyzed:

- **Working memory**, calculated as the number of visits that yielded a reward divided by the total number of visits (including revisits) to baited holes.
- **Reference memory**, calculated as the total number of visits to baited holes divided by the total number of visits to all holes. Reference memory was further divided into components reflecting spatial orientation and spatial pattern learning:
  ◦**Rotational reference memory** reflects rotational errors made while a pig was orienting himself after entering the holeboard, i.e. reference memory mistakes made prior to finding the first reward (35). This measure was calculated as 1 divided by the total number of visits to all holes up to and including the first rewarded visit.
  ◦**Spatial pattern reference memory** reflects a pig's ability to successfully complete the spatial pattern formed by the reward configuration, i.e., reference memory mistakes made after finding the first reward (36). This measure was calculated as total number of visits to baited holes divided by the total number of visits to all holes excluding visits made before finding the first reward.
- **Trial duration**, **latency to first visit** and **latency to first reward**, calculated as average time in seconds elapsed between entering the holeboard and performing the required action. When a pig failed to perform the required action, a maximum value of 450 s was assigned.
- **Inter-visit interval**, calculated as the average time in seconds between two successive visits.
- **Total number of visits, number of different locations (holes) visited** and **number of rewards found**, calculated as absolute counts.

Additionally, **trials to criterion** was calculated as the number of trials needed to reach criterion performance for the acquisition and first reversal phase.

### Cortisol analysis

#### Hair cortisol

Hair samples were collected at weaning and at the end of the experiment, when the pigs were ~5 months old. Hair was taken from the left flank of each pig with a razor (single edged disposable prep razor, Kai Medical, Solingen, Germany; a new razor was used for each sample). Hair cortisol concentration was determined based on the protocol by Davenport et al. (37). In short, samples were washed and dried, after which ~35 mg of hair was ground with a bead beater (TissueLyser II, QIAGEN Benelux B.V., Antwerp, Belgium) for a minimum of 2 × 15 min at 30 Hz, in 2 mL tubes containing three 2.3 mm steal beads (BioSpec, Lab Services B.V., Breda, the Netherlands). After grinding, 1 mL methanol was added and samples were incubated for 24 h with slow rotation to extract corticosteroids. Of the extract, 0.6 mL was dried using a vacuum centrifuge. Dried extracts were dissolved in 0.3 mL phosphate buffer. Hair cortisol concentrations were then determined in duplo using a Salimetrics Salivary Cortisol ELISA kit. Intra-assay and inter-assay coefficients of variation (CV) were 7.1 and 23.1%, respectively. The higher inter-assay CV implies plate-to-plate variation (i.e., different plates produced different cortisol concentrations for the same sample). To avoid an influence of inter-assay CV on group comparisons, samples were balanced across plates for birth weight and sex.

#### Salivary cortisol

Saliva samples were collected from each pig prior to and after their first individual habituation trial in the holeboard. Pre-stressor samples were collected at ~14:00 in the afternoon in their home pens. Post-stressor samples were taken ~20 min after a pig's trial in the holeboard, to allow for the peak in cortisol response to develop (38). Saliva was collected by allowing each pig to chew on two cotton swabs (Cotton Swabs 150 mm × 4 mm WA 2PL; Heinz Herenz, Hamburg, Germany) until they were sufficiently moistened. Then, the swabs were centrifuged using saliva collection tubes (Salivette, Sarstedt, Germany) at around 3,524 g for 10 min at 10°C. Saliva samples were stored at −20°C until salivary cortisol concentration was determined in duplo using a Coat-a-Count radioimmunoassay kit (Siemens Healthcare Diagnostics BV, The Hague, the Netherlands). Intra-assay and inter-assay CVs were 4.8 and 1.6%, respectively.

### Statistical analysis

All statistical analyses were performed using R statistical software, version 3.4.2 (39). For linear mixed models, package nlme (40) was used. For each mixed model the random effect structure was assessed using Restricted Maximum Likelihood (REML) estimation. Final selection of random effect structure was based on Akaike's information criterion (AIC). Round (first or second round of selected animals) did not improve fit of mixed models and was therefore dropped from further analysis. Statistical significance was set at *P* < 0.05. Effect size was calculated as Pearson's r based on contrasts. Unless indicated otherwise, results are presented as mean ± SEM.

#### Birth weight and growth

Average birth weight of LBW and NBW pigs was compared using Welch's *t*-test. The effect of birth weight on pigs' weekly weight gain from weaning until 5 months of age was analyzed using a linear mixed model with Birth weight, Week and Birth weight × Week interaction as fixed effects. Random effect structure consisted of random slopes and intercepts for Subject nested within Litter.

#### Holeboard data

For all variables scored during the acquisition and reversal phases, means of four successive trials (trial blocks) were calculated. Furthermore, to assess the effect of transitioning to a reversal phase, the last trial block of the acquisition phase was compared to the first trial block of the first reversal phase. The same was done for the transition from first to second reversal phase. The effect of birth weight on pigs' learning curves during acquisition, transition and reversal phases for all holeboard variables were analyzed using a linear mixed model with Birth Weight, Sex, Trial Block, and their two-way interactions as fixed effects. Random effect structure consisted of random intercepts for Subject nested within Litter and a first-order autoregressive correlation structure for residuals to account for repeated measures within subjects. The habituation phase was analyzed similarly, but with Trial as a fixed effect instead of Trial Block. Durations and latencies were log_10_ transformed to improve the distribution of residuals. Finally, trials to criterion for the acquisition and first reversal phase were compared using a linear mixed model with Birth Weight, Sex, and Birth Weight × Sex interaction as fixed effects and random intercepts for Litter.

#### Cortisol concentrations

The effects of birth weight on pigs' hair cortisol concentrations at weaning and 5 months of age were analyzed using a linear mixed model with Birth weight, Sex, and Birth weight × Sex interaction as fixed effects and random intercepts for Litter. Hair samples collected at weaning from three pigs (1 LBW male, 1 NBW male, and 1 NBW female) were insufficient for cortisol analysis. Therefore, hair cortisol analysis on samples at weaning was performed on the remaining 35 samples.

The effects of birth weight on salivary cortisol concentrations before and after a pig's first individual trial in the holeboard were analyzed using a linear mixed model with Birth Weight, Sex, Sample, and all two-way interactions as fixed effects and random slopes and intercepts for Subject. Salivary cortisol concentrations were log_10_ transformed to improve distribution of residuals. Saliva collected from one LBW male was insufficient for cortisol analysis. Therefore, salivary cortisol analysis was performed on samples collected from the remaining 37 animals.

## Results

### Birth weight and growth

**Figure 2:**
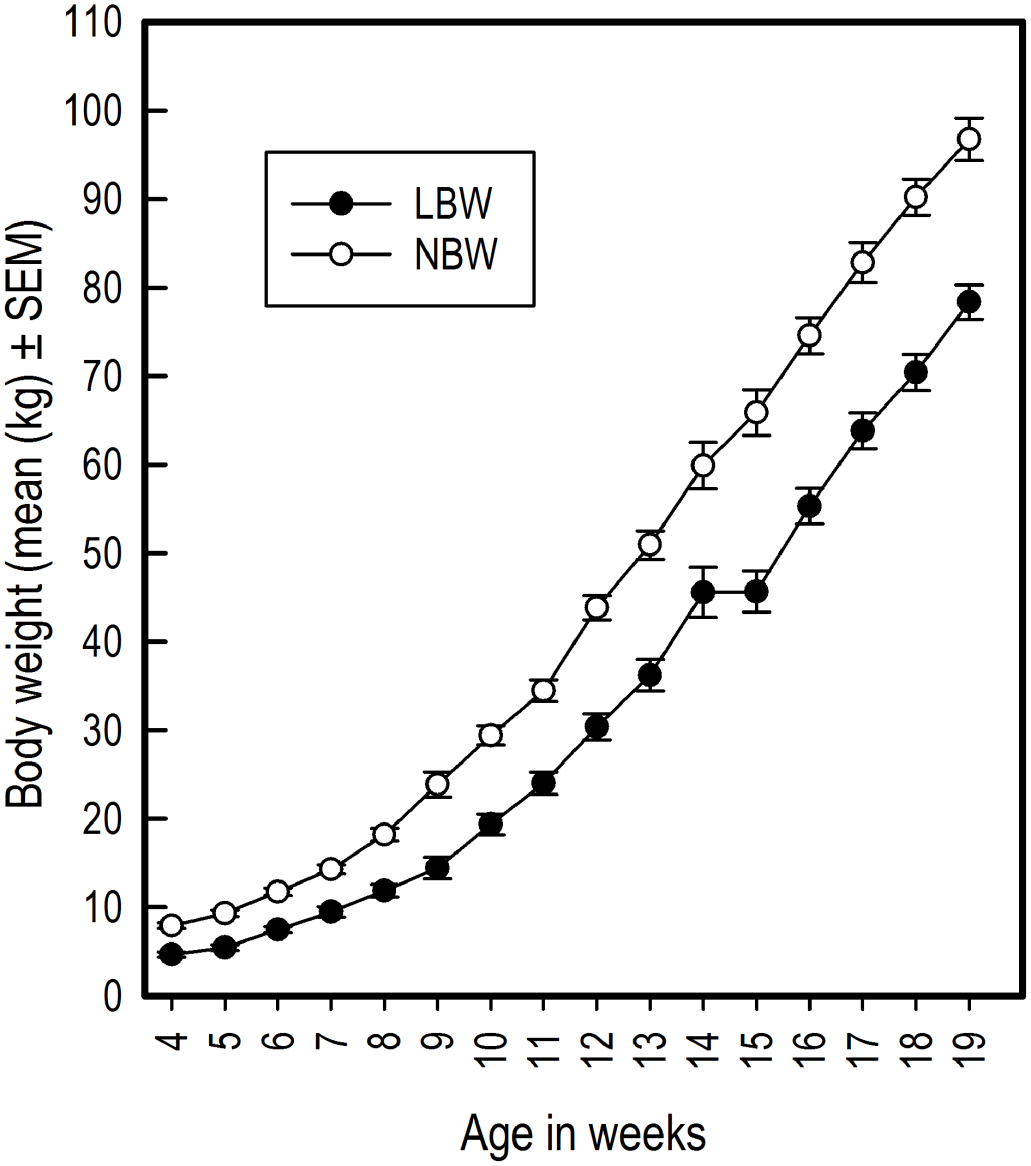
Average body weight in kilograms of LBW and NBW pigs from weaning until the end of the experiment. For week 15, data from only ten LBW pigs was available, causing an appearance of lack of growth from week 14 to 15. This is an artifact due to missing data.

LBW piglets had on average a lower birth weight than NBW piglets [LBW: 0.81 kg ± 0.02, NBW: 1.45 kg ± 0.05; *t*_(27.31)_ = −11.63, *P* < 0.001; *r* = 0.91]. LBW piglets continued to have lower body weight throughout the duration of the experiment [Birth weight: *F*_(1, 22)_ = 34.30, *P* < 0.001; *r* = 0.79; Figure 2) and had a slower growth rate than the NBW piglets [Birth weight × Week: *F*_(15, 482)_ = 10.39, *P* < 0.001; Figure 2).

### Spatial holeboard task

#### Habituation

Birth weight and sex did not influence pigs' performance during the habituation trials (Supplementary Table 1). During habituation, all pigs showed a comparable exploration of the holeboard based on total visits and locations (holes) visited.

#### Spatial learning and memory

##### Working memory

**Figure 3:**
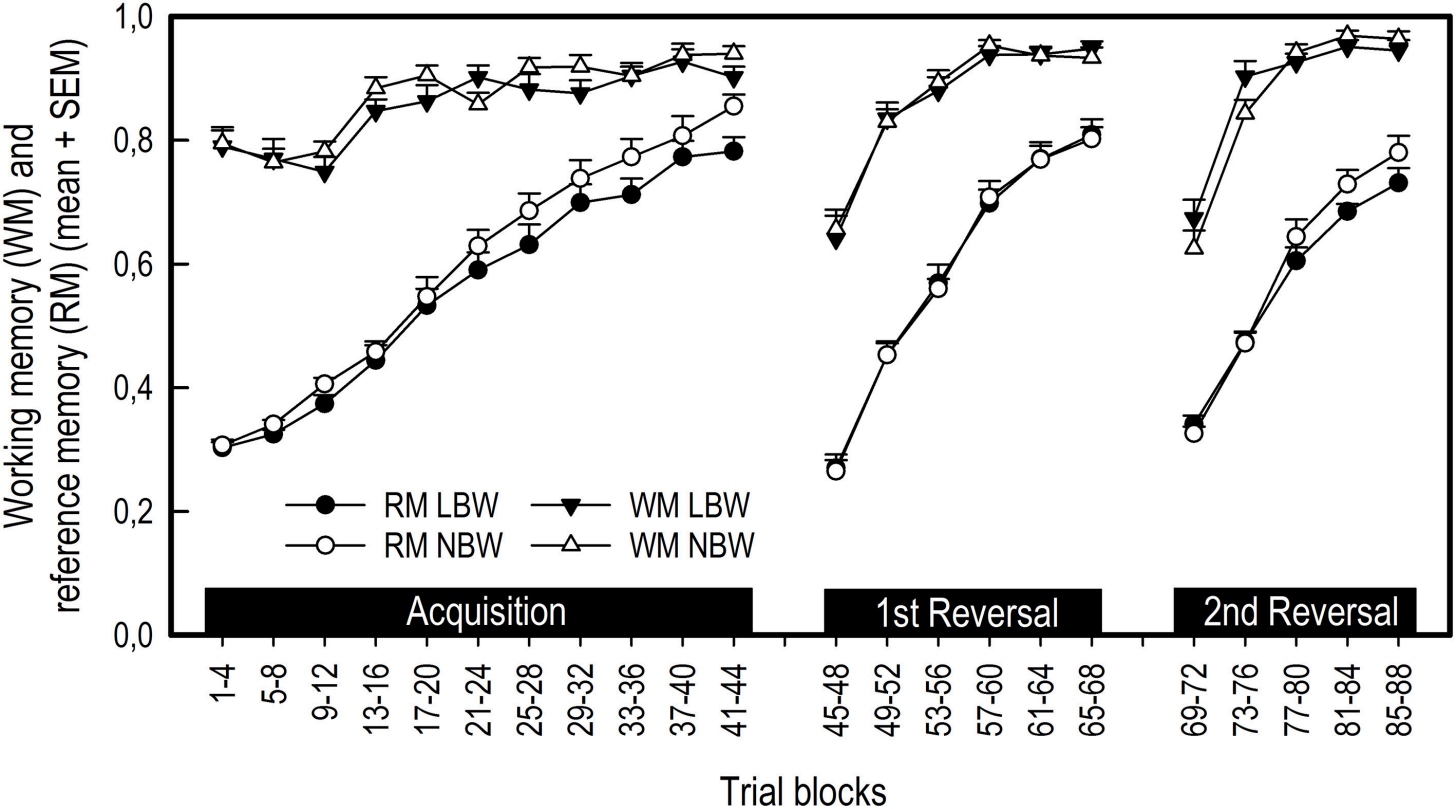
Average working memory (WM) and reference memory (RM) scores of LBW and NBW pigs in the spatial holeboard task per trial block. There was no significant effect of sex on holeboard performance, either as a main effect or interaction with birth weight. Therefore, the data for males and females have been combined.

Neither birth weight nor sex had an effect on working memory (WM) scores during any phase of the experiment (Figure 3; Table 2). All pigs improved their WM scores as training progressed during the acquisition phase, first reversal and second reversal [Trial blocks: Acquisition, *F*_(10, 350)_ = 16.13, *P* < 0.001; First reversal, *F*_(5, 174)_ = 55.39, *P* < 0.001; Second reversal, *F*_(4, 136)_ = 89.16, *P* < 0.001]. After a transition to a new configuration of baited holes, all pigs showed an initial decrease in WM scores [Trial blocks: First transition, *F*_(1, 34)_ = 139.19, *P* < 0.001; Second transition, *F*_(1, 34)_ = 175.11, *P* < 0.001].

**Table 2:** Spatial memory performance of low birth weight and normal birth weight pigs in the spatial holeboard task, during an acquisition (Acq), first transition (Trans I), first reversal (Rev I), second transition (Trans II), and second reversal (Rev II) phase.

	**Birth weight (BW)**			**Sex**			**BW** × **Sex**			**Trial blocks**			**BW** × **Trial blocks**			**Sex** × **Trial blocks**		
**Measure Phase**	***F***	***df***	***P ≤***	***F***	***df***	***P ≤***	***F***	***df***	***P ≤***	***F***	***df***	***P ≤***	***F***	***df***	***P ≤***	***F***	***df***	***P ≤***
**WM**																		
Acq	2.22	1,20	0.152	0.18	1,20	0.674	1.63	1,20	0.217	**16.13**	**10,350**	<**0.001**	1.03	10,350	0.422	1.25	10,350	0.258
Trans I	0.08	1,20	0.780	0.47	1,20	0.450	1.45	1,20	0.242	**139.19**	**1,34**	<**0.001**	0.56	1,34	0.459	0.06	1,34	0.803
Rev I	0.02	1,20	0.894	0.00	1,20	< 0.999	0.37	1,20	0.549	**55.39**	**5,174**	<**0.001**	0.20	5,174	0.964	0.39	5,174	0.855
Trans II	1.45	1,19	0.243	0.03	1,19	0.858	*3.32*	*1,19*	*0.084*	**175.11**	**1,34**	<**0.001**	1.22	1,34	0.277	0.01	1,34	0.934
Rev II	0.73	1,19	0.402	0.16	1,19	0.691	0.46	1,19	0.505	**89.16**	**4,136**	<**0.001**	*2.01*	*4,136*	*0.096*	0.34	4,136	0.854
**RM**																		
Acq	**5.76**	**1,20**	**0.026**	1.03	1,20	0.322	0.93	1,20	0.344	**104.71**	**10,350**	<**0.001**	0.58	10,350	0.832	1.04	10,350	0.407
Trans I	0.21	1,20	0.649	0.00	1,20	0.978	0.01	1,20	0.931	**1787.92**	**1,35**	<**0.001**	0.08	1,35	0.783	0.37	1,35	0.549
Rev I	0.01	1,20	0.926	0.66	1,20	0.427	0.01	1,20	0.920	**173.34**	**5,175**	<**0.001**	0.07	5,175	0.996	0.86	5,175	0.508
Trans II	0.00	1,19	0.976	0.54	1,19	0.470	0.00	1,19	0.979	**1882.18**	**1,34**	<**0.001**	1.63	1,34	0.210	0.30	1,34	0.589
Rev II	1.65	1,19	0.214	1.75	1,19	0.201	0.24	1,19	0.633	**54.74**	**4,136**	<**0.001**	1.10	4,136	0.362	*2.35*	*4,136*	*0.058*
**rRM**																		
Acq	0.45	1,20	0.509	0.23	1,20	0.640	0.10	1,20	0.758	**49.90**	**10,350**	<**0.001**	**2.04**	**10,350**	**0.029**	0.73	10,350	0.695
Trans I	0.10	1,34	0.749	1.23	1,34	0.276	0.08	1,34	0.778	**567.30**	**1,35**	<**0.001**	**5.27**	**1,35**	**0.028**	**4.15**	**1,35**	**0.049**
Rev I	2.54	1,20	0.126	0.86	1,20	0.366	0.83	1,20	0.374	**82.78**	**5,175**	<**0.001**	1.34	5,175	0.249	1.57	5,175	0.172
Trans II	0.48	1,19	0.497	*3.26*	*1,19*	*0.087*	1.43	1,19	0.246	**164.70**	**1,34**	<**0.001**	0.05	1,34	0.829	0.00	1,34	0.945
Rev II	1.63	1,19	0.217	0.01	1,19	0.914	1.42	1,19	0.248	**43.10**	**4,136**	<**0.001**	0.03	4,136	0.999	0.41	4,136	0.804
**sRM**																		
Acq	**5.51**	**1,20**	**0.029**	1.22	1,20	0.283	1.24	1,20	0.279	**108.61**	**10,350**	<**0.001**	0.60	10,350	0.811	0.97	10,350	0.472
Trans I	0.08	1,20	0.782	1.38	1,20	0.254	1.72	1,20	0.204	**1198.26**	**1,35**	<**0.001**	0.33	1,35	0.569	0.00	1,35	0.988
Rev I	0.22	1,20	0.647	0.10	1,20	0.763	0.04	1,20	0.850	**140.71**	**5,175**	<**0.001**	0.23	5,175	0.948	1.06	5,175	0.385
Trans II	0.01	1,19	0.906	0.40	1,19	0.533	0.75	1,19	0.396	**977.78**	**1,34**	<**0.001**	1.35	1,34	0.254	0.67	1,34	0.419
Rev II	1.30	1,19	0.269	1.59	1,19	0.222	0.16	1,19	0.697	**152.19**	**4,136**	<**0.001**	1.21	4,136	0.310	**2.97**	**4,136**	**0.022**

*Effects printed in bold have associated probabilities of < 0.05. Effects printed in italics have associated probabilities of 0.10 > P ≥ 0.05. WM, working memory; RM, reference memory; rRM, rotational reference memory; sRM, spatial pattern reference memory*.

##### Reference memory

Birth weight affected reference memory (RM) scores during the acquisition phase [Birth weight: *F*_(1, 20)_ = 5.76, *P* = 0.026, *r* = 0.12; Figure 3; Table 2), with LBW piglets scoring lower than NBW piglets. This difference was transient, with no effect of birth weight on RM scores during either the first or second reversal phase [Birth weight: First reversal, *F*_(1, 20)_ = 0.01, *P* = 0.926; Second reversal, *F*_(1, 19)_ = 1.65, *P* = 0.214]. Similarly, there was a trend for LBW piglets to require a higher number of trials to complete the acquisition phase compared to NBW piglets [LBW: 53.26 ± 2.27, NBW: 48.00 ± 1.70; *F*_(1, 20)_ = 4.19, *P* = 0.054]. No effect of birth weight was found on the number of trials required to complete the first reversal phase [LBW: 29.26 ± 1.38, NBW: 29.79 ± 1.08; *F*_(1, 20)_ = 0.08, *P* = 0.776]. Sex had no effect on trials to criterion during the acquisition phase [Sex: *F*_(1, 20)_ = 2.46, *P* = 0.132; Sex × Birth weight: *F*_(1, 20)_ = 0.46, *P* = 0.504] or the first reversal phase [Sex: *F*_(1, 20)_ = 0.08, *P* = 0.775; Sex × Birth weight: *F*_(1, 20)_ = 1.41, *P* = 0.249], nor did it influence RM scores during any phase of the experiment (Table 2). All pigs improved their RM scores during the acquisition, first reversal and second reversal phases [Trial blocks: Acquisition, *F*_(10, 350)_ = 104.71, *P* < 0.001; First reversal, *F*_(5, 175)_ = 173.34, *P* < 0.001; Second reversal, *F*_(4, 136)_ = 54.74, *P* < 0.001]. After each transition to a reversal phase, all pigs showed an initial decrease in RM scores [Trial blocks: First transition, *F*_(1, 35)_ = 1787.92, *P* < 0.001; Second transition, *F*_(1, 34)_ = 1882.18, *P* < 0.001].

RM scores can be separated into a rotational and a spatial pattern component. Birth weight influenced rotational RM (rRM) scores for certain specific trial blocks during the acquisition phase and transition to the first reversal phase, as indicated by Birth weight × Trial blocks interactions. For the acquisition phase, LBW pigs had lower rRM scores for trials 21–24 and 25–28 [Birth weight × Trial blocks: *F*_(10, 350)_ = 2.04, *P* = 0.029]. For the first transition phase, LBW pigs had higher rRM scores for the final trial block of the acquisition phase and lower rRM scores for the first trial block of the first reversal phase [Birth weight × Trial blocks: *F*_(1, 35)_ = 5.27, *P* = 0.028]. Together, these findings do not represent a systematic difference between LBW and NBW pigs for rRM scores (Table 2). However, a general effect of birth weight was found for spatial pattern RM scores during the acquisition phase [Birth weight: *F*_(1, 20)_ = 5.51, *P* = 0.029, *r* = 0.12], suggesting the difference found between LBW and NBW pigs in RM performance reflects a difference in spatial pattern learning. No effect of birth weight on subsequent phases was found [Birth weight: First reversal, *F*_(1, 20)_ = 0.22, *P* = 0.647; Second reversal, *F*_(1, 19)_ = 1.30, *P* = 0.269]. A difference between female and male pigs was only found for certain trial blocks during the transition from acquisition to first reversal phase and during the second reversal phase. Male pigs had higher rRM scores for the final trial block of the acquisition phase [Sex × Trial blocks: *F*_(1, 35)_ = 4.15, *P* = 0.049] and higher sRM scores for the final trial block of the second reversal phase [Sex × Trial blocks: *F*_(4, 136)_ = 2.97, *P* = 0.022]. Similar to the general RM scores, both rotational and spatial pattern RM scores improved within training phases, but initially decreased when pigs were transitioned to a reversal phase (Table 2).

#### Duration measures

Birth weight had an effect on the latency to first reward during the acquisition phase, with LBW pigs taking longer than NBW pigs to find their first reward [Birth weight: *F*_(1, 20)_ = 6.40, *P* = 0.012, *r* = 0.35]. This finding was due to a difference between groups for the first trial blocks and thus does not reflect a systematic difference in performance between LBW and NBW pigs. Similarly, female pigs had higher inter-visit intervals compared to male pigs during the first trial block of the second reversal phase [Sex × Trial blocks: *F*_(4, 136)_ = 4.07, *P* = 0.004]. No other effects of birth weight or sex on duration measures were found (Trial duration, Latency to first visit, Latency to first reward and Inter-visit interval; Supplementary Table 1). Most duration measures decreased as training progressed during the acquisition, first reversal and second reversal phase, with pigs needing less time to finish a trial. Latency to first visit increased during the acquisition phase, likely due to pigs learning to approach a rewarded location for their first visit, instead of simply visiting the nearest hole upon entering the holeboard [Trial block: Acquisition, *F*_(10, 350)_ = 13.35, *P* < 0.001]. Most duration measure scores initially increased after a transition to a reversal. The only exception was latency to first visit, which remained stable after the start of both reversal phases [Trial block: First transition, *F*_(1, 35)_ = 3.09, *P* = 0.087; Second transition, *F*_(1, 34)_ = 0.26, *P* = 0.615]. As the second reversal progressed, only a trend for an increase in latency to first visit was found [Trial block: Second reversal, *F*_(4, 136)_ = 2.33, *P* = 0.059].

#### Exploration measures

No systematic effects of birth weight or sex were found for any of the exploration measures assessed (Total number of visits, Number of locations visited and Number of rewards found). LBW pigs found less rewards than NBW pigs during the first trial blocks of the acquisition phase [Birth weight × Trial blocks: *F*_(10, 350)_ = 2.13, *P* = 0.022]. Female pigs visited more locations than male pigs during the final trial blocks of the second reversal phase [Sex × Trial blocks: *F*_(4, 136)_ = 2.84, *P* = 0.027]. No other effects of birth weight or sex were found. Scores for all exploration measures improved as pigs progressed during the acquisition, first reversal and second reversal phases (Supplementary Table 1). As training progressed, pigs required fewer total visits, visited fewer locations, and found a higher number of rewards. The opposite was true when pigs were subjected to the reversal phases.

### Cortisol concentrations

#### Hair cortisol

At weaning, cortisol concentration in flank hair of LBW piglets was higher than that of NBW piglets [LBW: 33.20 ± 1.68, NBW: 29.26 ± 1.41; *F*_(1, 18)_ = 5.38, *P* = 0.032, *r* = 0.34]. Sex did not influence hair cortisol concentration at weaning [Sex: *F*_(1, 18)_ = 0.00, *P* = 0.975; Sex × Birth weight: *F*_(1, 18)_ = 0.04, *P* = 0.847]. The difference between birth weight categories was no longer present in hair samples collected at 5 months of age, at the end of the experiment [LBW: 20.61 ± 1.14, NBW: 21.84 ± 1.88; *F*_(1, 20)_ = 0.33, *P* = 0.575]. Again, sex did not influence hair cortisol concentration [Sex: *F*_(1, 20)_ = 2.96, *P* = 0.101; Sex × Birth weight: *F*_(1, 20)_ = 0.52, *P* = 0.480].

#### Salivary cortisol

**Figure 4:**
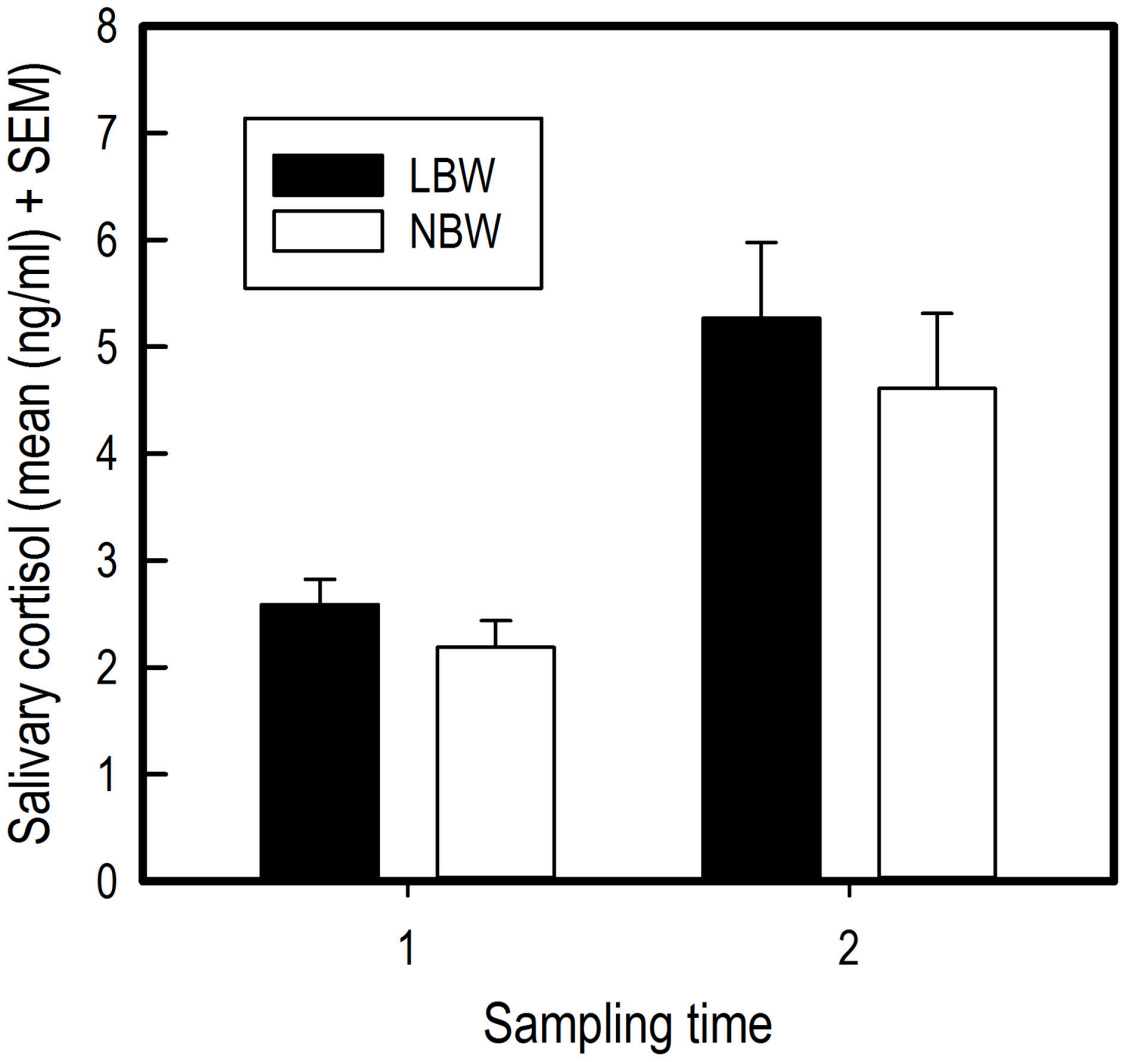
Average salivary cortisol concentrations of LBW and NBW pigs before and after a stressor. There was no significant effect of sex on cortisol concentrations, either as a main effect or interaction with birth weight. Therefore, the data for males and females have been combined.

Performing the first individual trial in the spatial holeboard task caused an increase in salivary cortisol concentration for all piglets [*F*_(1, 34)_ = 31.53, *P* < 0.001; Figure 4). No effects of birth weight [Birth weight: *F*_(1, 33)_ = 2.55, *P* = 0.120; Birth weight × Sample: *F*_(1, 34)_ = 0.01, *P* = 0.924; Figure 4) or sex [Sex: *F*_(1, 33)_ = 1.15, *P* = 0.292; Sex x Sample: *F*_(1, 34)_ = 0.18, *P* = 0.677; Sex x Birth weight: *F*_(1, 33)_ = 1.21, *P* = 0.279] were found on salivary cortisol concentrations.

## Discussion

The present study assessed the effects of LBW on post-weaning cognitive performance in pigs. To this end, it is important that the LBW piglets selected for our study actually represented a different population than the selected piglets with NBW. Indeed, LBW piglets had significantly lower birth weights than NBW piglets. Furthermore, the LBW piglets selected for our study had birth weights comparable to or smaller than those of piglets assessed in other LBW studies [e.g., (18, 19, 22)]. In pigs, birth weight is the main measure used to determine whether intra-uterine growth restriction has occurred (41, 42). Head morphology has been suggested as an additional measure, where a relatively large head is considered a sign of the so-called “brain sparing effect”, i.e., placental insufficiency resulting in prioritized brain development (22, 43). However, head morphology has been shown to correlate with birth weight (43) and can also be confounded by differences in head shape between different pig breeds (own, non-systematic observations). Therefore, birth weight remains the best indicator that the LBW pigs used in our study suffered from limited nutrients and oxygen *in utero*. The found difference in body weight of LBW and NBW pigs persisted throughout the duration of the experiment. This shows LBW pigs continued to experience impaired weight gain well beyond weaning. Long-term effects on growth have also been shown in previous studies with LBW pigs [e.g., (22, 44)]. Such a lack of catch-up growth shown by LBW offspring is considered an additional risk factor for cognitive impairment in humans (6).

Based on previous studies assessing the effects of LBW in both humans and pigs [e.g., (6, 17, 18)], it was expected that LBW pigs would have an impaired cognitive development as shown by lower memory scores in the spatial holeboard task compared to NBW pigs. Both groups of pigs were able to acquire the task, producing similar learning curves to previous holeboard studies with pigs [e.g. (23, 45)]. Pigs improved their performance as training progressed, as shown by increasing memory scores and decreasing latencies and exploration. In line with our expectation, LBW pigs had lower reference memory (RM) scores during the acquisition phase of the experiment. Additionally, LBW pigs had higher average hair cortisol concentrations (HCC) than NBW pigs in samples taken at weaning. This implies LBW pigs experienced more chronic stress during pre-weaning development. Both the cognitive impairment and the increased HCC found for LBW pigs were transient, likely due to the enriched housing conditions applied during this study.

### Effects of LBW on spatial learning and memory

Birth weight was found to cause a mild cognitive impairment, based on spatial learning and memory in the holeboard task. Compared to NBW pigs, LBW pigs showed lower RM scores as the acquisition phase of the experiment progressed. This finding indicates that LBW pigs had more difficulty learning and remembering the locations of food rewards in the holeboard. It is unlikely this effect of LBW was caused by a difference in motivation between LBW and NBW pigs to perform the task. Both groups showed comparable scores on measures of motivation, such as the latency to first visit and the inter-visit interval. This is corroborated by an earlier study comparing food motivation of LBW and NBW pigs (46).

Our finding of impaired cognitive development is supported by earlier studies showing decreased learning and memory associated with LBW in pigs (17, 18). Similarly, LBW in humans has been shown to cause learning difficulties throughout adolescence (6, 7), as well as impaired spatial learning (47). However, there have also been studies which have shown LBW pigs to have a comparable, or even superior, cognitive performance compared to NBW pigs (19–22). Several factors could have contributed to this discrepancy in results.

First, it is difficult to compare the results found by Antonides *et al*. (who reported improved cognitive performance of LBW pigs) to those of other studies assessing post-weaning cognition in LBW pigs, including the current study. This is due to large differences in housing conditions. Their pigs were removed from the sow at 4–6 days of age, whereas other studies applied weaning at 4 weeks of age, comparable to standard commercial practice. Abrupt changes in neonatal environment have been shown to impact piglet development, resulting in increased behavioral and physiological signs of stress (48, 49). Additionally, there was a considerable difference in stocking density. Antonides *et al*. provided 0.625–1.25 m^2^ space per pig, whereas the other studies provided a minimum of ~2 m^2^ per pig (18–21). A higher stocking density affects pig welfare mainly through increased aggression (50). This could have impacted NBW pigs more, as they remain larger than LBW pigs. Taken together, these differences in housing conditions may have influenced the pigs' early development, hindering direct comparison of results.

Second, the findings of previous studies examining LBW pigs have all been based on smaller sample sizes than applied in our study. Smaller sample sizes increase the probability of chance findings (51), potentially leading to contradictory results in replication studies. Other factors influencing cognitive abilities, e.g., personality (52), could then lead to a significant difference between groups that does not reflect the effects of birth weight. In particular, several studies reporting comparable cognitive performance of NBW and LBW pigs have based their results on relatively small sample sizes. For example, Murphy and colleagues (20) compared six NBW to five LBW pigs in a conditional discrimination task, where both groups were equally capable of learning the task. Similarly, Gieling and colleagues (19) found comparable spatial holeboard performance of LBW and NBW pigs by using litter as the experimental unit in data analysis (i.e., average performance of LBW or NBW litter mates was analyzed instead of individual performance of each pig). This resulted in a loss of statistical power by reducing the effective sample size. Interestingly, visual inspection of the RM scores of their pigs show a similar pattern to the current study. Control LBW pigs (half of the animals were prenatally treated with an anti-oxidative drug) have lower average RM scores toward the end of the acquisition phase.

Finally, it is possible that LBW has not consistently been found to impair cognition in pigs due to the use of different cognitive tasks in different studies. Cognitive development of LBW pigs has been assessed using measures of spatial learning (17–19, 22) and associative learning (20, 21). Spatial learning in a holeboard task and associative learning in a conditional discrimination task have previously been found to be independent measures of cognition in pigs (53). Perhaps no effects of LBW were found in associative learning studies with pigs because they assessed a cognitive domain that is less vulnerable to impairment as a result of LBW. Such specific effects of LBW, with varying effects on different cognitive tasks, have previously been reported for humans (54) and rats (55). That LBW does not have a general negative effect on cognitive development in pigs is also supported by our finding of decreased RM, but not working memory (WM) scores. This is corroborated by earlier holeboard studies (18, 19, 22), where LBW and NBW pigs show very comparable WM learning curves during the acquisition of the holeboard task [although one study found an effect of LBW on WM scores during reversal learning, implying impaired behavioral flexibility—(18)]. Furthermore, when separating the general RM scores into rotational RM scores based on the ability of orientation within the environment (35) and spatial pattern RM scores based on spatial pattern learning (36), our results show that birth weight only affected spatial pattern RM scores. After entering the holeboard, LBW and NBW pigs were equally capable of orienting themselves and locating a baited hole. However, completing the spatial pattern of baited holes after finding this first reward was impaired in LBW pigs. This provides further evidence that LBW could have specific effects on different cognitive domains. It would be relevant for future studies to assess the effects of LBW on additional cognitive domains in pigs, mainly those that are relevant for their welfare. For example, as social behaviors are of such importance to pig welfare (13), testing the effects of LBW on social cognition is recommended.

### Effects of LBW on pre-weaning chronic stress

Previous studies with pigs have shown that LBW causes an altered functioning of the HPA axis. LBW pigs show increased baseline cortisol levels, both pre- and post-weaning (27, 56). Furthermore, LBW pigs show an exaggerated acute stress response (29). It was therefore expected that the LBW pigs in our study would suffer from a similar increase in HPA axis activity, namely an increase in hair cortisol concentration compared to NBW pigs, indicative of chronic stress and an exaggerated increase in salivary cortisol compared to NBW pigs after exposure to an acute stressor. These expectations were only partially confirmed.

HCC was used as a non-invasive measure of chronic stress (57). As cortisol is incorporated into the growing hair shaft, HCC allows for assessment of HPA axis activity over a longer time period than other biomarkers of stress. HCC as a measure of chronic stress has previously been assessed in pigs [e.g., (22, 58)]. For example, barren housing leads to a higher HCC in pigs (58). These results are comparable to chronic stress assessment using HCC in other species, with long-term stress leading to increased HCC (37).

At weaning, LBW pigs showed an increased HCC compared to NBW pigs. This suggests that LBW pigs experienced more chronic stress while in the farrowing pens. It is known that LBW piglets experience more physiological stressors after farrowing. For example, LBW piglets suffer from impaired thermoregulation (59) and are less likely to acquire a (desirable) teat when suckling (60) compared to their NBW siblings. However, in our study the effects of such stressors were mitigated by providing extra heating in the farrowing pens (both heat lamps and floor heating), as well as applying cross-fostering to ensure the number of piglets in a litter did not exceed the sow's number of teats. It is possible that in addition to the increased physiological stressors, LBW piglets display an exaggerated response to chronic environmental stressors. Housing piglets in farrowing pens, in which the sow is constrained in a farrowing crate, can lead to behavioral signs of decreased welfare, such as decreased play behavior (61). This could be due to the lack of space and limited opportunities for sow-piglet interactions (62). Based on our results, LBW piglets are more chronically stressed in the farrowing environment than their NBW siblings. Future studies are required to establish which specific physiological or environmental stressors are responsible for LBW piglets' chronic stress.

### Ameliorating effects of enrichment

Only a mild impairment of spatial cognition was found, along with a transient increase in chronic stress. It is likely that the effects of LBW found were ameliorated by the enriched housing conditions applied during this study.

Enriched housing has been shown to improve cognitive performance in pigs (34, 63). Furthermore, it has been suggested that training animals—in particular if training extends over a longer time period—may act as cognitive enrichment (64). Together, the environmental and cognitive enrichment applied in the current study may have alleviated the effects of adverse conditions, such as LBW. Therefore, it is possible that the LBW pigs in our study performed better than they would have done if they had been housed in the barren conditions that are standard practice on most commercial farms. Future studies exploring the post-weaning cognitive abilities of LBW pigs in different housing conditions are encouraged.

Several indications were found that enrichment also had an ameliorating effect on LBW pigs' stress response. First, in contrast to pre-weaning HCC, post-weaning HCC was not influenced by birth weight. This suggests both groups of pigs were experiencing similar levels of chronic stress once they were moved to the research facilities. Previous studies have shown enriched housing conditions decrease stress as measured by HCC and serum cortisol concentration in pigs (58, 65). Therefore, it is likely that in our study, neither LBW nor NBW pigs experienced chronic stress after weaning. Similar findings have been reported by Murphy *et al*., who compared mean salivary cortisol concentration (SCC) and found no difference between LBW and NBW pigs housed in enriched conditions [(21), cf. (22)].

Second, we found no exaggerated acute stress response in LBW pigs. In our study, SCC was used as a non-invasive measure of acute stress (66). Both LBW and NBW pigs showed increased SCC after performing the first individual trial in the holeboard, indicating the applied stressor was successful (38). However, this increase in SCC was similar for LBW and NBW pigs. This provides further suggestion that the enrichment applied during our study had an ameliorating effect on LBW pigs' stress response, as a previous study has found LBW pigs to show an exaggerated response to acute stress (29). Future research aimed at the comparison of HPA axis functioning between LBW and NBW pigs housed in standard commercial conditions is encouraged. A longitudinal study of hair cortisol on commercial pig farms should be feasible, as collection of samples is non-invasive. The acute stress response of LBW and NBW pigs could be compared by collecting saliva samples prior to and after common stressors on commercial farms, such as ear tagging and tail docking at a few days old or mixing animals after weaning.

### No sex effects on cognition or cortisol

Our study is the first to control for an effect of sex on the cognitive development of LBW pigs. This was done to account for a possible sex-dependent effect of stress on learning and memory, as has been found in other species (25, 26). Such effects were expected to be exaggerated in LBW pigs, due to their altered HPA-axis functioning (28–30, 58). However, sex did not systematically influence any of the measures for spatial learning and memory in the holeboard, possibly because we failed to find an exaggerated stress response in our LBW pigs (see section Ameliorating Effects of Enrichment).

In a previous study examining the effects of sex on spatial holeboard performance, male pigs showed impaired behavioral flexibility when faced with a reversal of the task (23). This result was not corroborated in the current study. Independent of birth weight, female and male pigs showed a similar exploration of the holeboard and were equally able to find the rewarded holes during the reversal phase. These contrasting results could be due to a difference in housing conditions between the two studies, with the previous study housing females and males separately. In our study, pigs were grouped according to birth weight category, resulting in mixed-sex groups. The effects of mixed- vs. single-sex housing in pigs in relation to their behavior and stress response has not yet received extensive scientific attention. Whether being housed in a mixed-sex group is more or less stressful may differ for males and females, as male pigs have been shown to engage in more aggressive behaviors than females (67, 68). It has also been shown that male aggression is provoked more in mixed-sex groups than when males are housed separately from females (68). As group composition appears to influence aggressive interactions and thereby social stress (69), it is possible that it also impacts pigs' behavioral flexibility.

## Conclusion

Our results show that LBW causes a transient cognitive impairment in weaned pigs, as measured by a spatial holeboard task. An impaired development of spatial cognition could have adverse effects on the welfare of LBW pigs, as they require spatial learning and memory to correctly respond to their environment. For example, remembering specific locations, such as food and water sites, preferred areas for resting or the preferred areas of dominant conspecifics, is relevant to pig welfare (70, 71). LBW pigs also showed a transient increase in HCC, implying increased chronic stress in the farrowing environment. It is likely the effects of LBW found were mitigated by the enriched housing conditions applied during this study. Therefore, future studies assessing the cognitive development and stress responses of LBW pigs in commercial housing conditions are encouraged.

## Author contributions

FvdS, RN, and SR contributed to conception and design of the study. IvB, SM, and SR contributed to data acquisition. SR performed statistical analysis and wrote the first draft of the manuscript. All authors contributed to manuscript revision and have read and approved the submitted version.

### Conflict of interest statement

The authors declare that the research was conducted in the absence of any commercial or financial relationships that could be construed as a potential conflict of interest.
