# Supplementary material for: Low Birth Weight Impairs Acquisition of Spatial Memory Task in Pigs
Source: Front Vet Sci. 2018 Jun 26;5:142. doi: 10.3389/fvets.2018.00142 (PMC6028702; doi:10.3389/fvets.2018.00142)
Supplement: Supplementary file 1 [file Table_1.DOCX]

Supplementary Material

Low birth weight impairs acquisition of spatial memory task in pigs

Sanne Roelofs^*^, Ilse van Bommel, Stephanie Melis, Franz Josef van der Staay, Rebecca E. Nordquist

*** Correspondence:** Sanne Roelofs: [sanneroelofs@gmail.com](mailto:sanneroelofs@gmail.com)

**Supplementary Table 1.** Performance of low birth weight and normal birth weight piglets in the spatial holeboard task, during a habituation (Hab), acquisition (Acq), first transition (Trans I), first reversal (Rev I), second transition (Trans II) and second reversal (Rev II) phase. Effects printed in bold have associated probabilities of <0.05. Effects printed in italics have associated probabilities of 0.10 > P ≥ 0.05.

|  |  | **Birth weight (BW)** | | | **Sex** | | | **BW x Sex** | | | **Trial blocks** | | | **BW x Trial blocks** | | | **Sex x Trial blocks** | | |
| --- | --- | --- | --- | --- | --- | --- | --- | --- | --- | --- | --- | --- | --- | --- | --- | --- | --- | --- | --- |
|  | **Measure** |  |  |  |  |  |  |  |  |  |  |  |  |  |  |  |  |  |  |
|  | Phase | F | df | P≤ | F | df | P≤ | F | df | P≤ | F | df | P≤ | F | df | P≤ | F | df | P≤ |
|  |  |  |  |  |  |  |  |  |  |  |  |  |  |  |  |  |  |  |  |
|  | **TD** |  |  |  |  |  |  |  |  |  |  |  |  |  |  |  |  |  |  |
|  | Hab* | *3.27* | *1,20* | *.086* | 0.08 | 1,20 | .784 | *3.01* | *1,20* | *.098* | **5.14** | **3,105** | **.002** | 0.15 | 3,105 | .930 | 1.28 | 3,105 | .286 |
|  | Acq | *3.34* | *1,20* | *.083* | 0.81 | 1,20 | .378 | 0.50 | 1,20 | .487 | **35.19** | **10,350** | **<.001** | 0.84 | 10,350 | .586 | 1.22 | 10,350 | .275 |
|  | Trans I | 0.29 | 1,20 | .594 | 0.28 | 1,20 | .606 | 0.10 | 1,20 | .751 | **858.47** | **1,35** | **<.001** | 0.14 | 1,35 | .707 | 0.04 | 1,35 | .840 |
|  | Rev I | 0.14 | 1,20 | .711 | 0.05 | 1,20 | .835 | 0.12 | 1,20 | .739 | **142.47** | **5,175** | **<.001** | 0.26 | 5,175 | .934 | 0.56 | 5,175 | .730 |
|  | Trans II | 0.07 | 1,19 | .798 | 0.58 | 1,19 | .455 | *3.47* | *1,19* | *.078* | **577.78** | **1,34** | **<.001** | 0.60 | 1,34 | .443 | 1.18 | 1,34 | .285 |
|  | Rev II | 0.79 | 1,19 | .385 | 1.14 | 1,19 | .230 | 0.60 | 1,19 | .450 | **109.61** | **4,136** | **<.001** | *2.34* | *4,136* | *.058* | 1.98 | 4,136 | .101 |
|  | **LFV** |  |  |  |  |  |  |  |  |  |  |  |  |  |  |  |  |  |  |
|  | Hab* | *3.80* | *1,20* | *.065* | 0.00 | 1,20 | <.999 | 1.79 | 1,20 | .196 | **5.58** | **3,105** | **.001** | 0.17 | 3,105 | .917 | 0.79 | 3,105 | .504 |
|  | Acq | 1.04 | 1,20 | .320 | 0.48 | 1,20 | .498 | 0.01 | 1,20 | .913 | **13.35** | **10,350** | **<.001** | 1.55 | 10,350 | .120 | 0.92 | 10,350 | .512 |
|  | Trans I | 0.42 | 1,20 | .524 | 0.04 | 1,20 | .850 | 0.11 | 1,20 | .741 | *3.09* | *1,35* | *.087* | 0.04 | 1,35 | .850 | 0.00 | 1,35 | .994 |
|  | Rev I | 0.35 | 1,20 | .559 | 0.16 | 1,20 | .696 | 0.06 | 1,20 | .802 | **8.53** | **5,175** | **<.001** | 0.23 | 5,175 | .949 | 0.75 | 5,175 | .586 |
|  | Trans II | 0.19 | 1,19 | .664 | 0.20 | 1,19 | .658 | 1.04 | 1,19 | .320 | 0.26 | 1,34 | .615 | 0.06 | 1,34 | .803 | 0.98 | 1,34 | .329 |
|  | Rev II | 0.27 | 1,19 | .607 | 0.22 | 1,19 | .646 | 0.75 | 1,19 | .398 | *2.33* | *4,136* | *.059* | 0.31 | 4,136 | .868 | 1.48 | 4,136 | .210 |
|  |  |  |  |  |  |  |  |  |  |  |  |  |  |  |  |  |  |  |  |
|  | **IVI** |  |  |  |  |  |  |  |  |  |  |  |  |  |  |  |  |  |  |
|  | Hab* | *3.08* | *1,20* | *.095* | 0.09 | 1,20 | .761 | *3.39* | *1,20* | *.081* | **5.28** | **3,105** | **.002** | 0.40 | 3,105 | .750 | 0.39 | 3,105 | .758 |
|  | Acq | 2.84 | 1,20 | .108 | *3.13* | *1,20* | *.092* | 1.32 | 1,20 | .264 | **5.20** | **10,350** | **<.001** | *1.83* | *10,350* | *.054* | 0.68 | 10,350 | .739 |
|  | Trans I | 1.43 | 1,20 | .246 | 0.01 | 1,20 | .917 | 0.00 | 1,20 | .961 | **33.48** | **1,35** | **<.001** | 0.66 | 1,35 | .423 | 0.01 | 1,35 | .939 |
|  | Rev I | 0.60 | 1,20 | .448 | 0.00 | 1,20 | .973 | 0.00 | 1,20 | .976 | **39.46** | **5,175** | **<.001** | 0.76 | 5,175 | .583 | 0.46 | 5,175 | .804 |
|  | Trans II | 0.10 | 1,19 | .756 | 2.04 | 1,19 | .170 | 0.07 | 1,19 | .798 | **24.78** | **1,34** | **<.001** | 0.36 | 1,34 | .551 | *4.01* | *1,34* | *.053* |
|  | Rev II | 0.04 | 1,19 | .845 | 1.55 | 1,19 | .228 | 0.21 | 1,19 | .650 | **18.78** | **4,136** | **<.001** | 1.81 | 4,136 | .130 | **4.07** | **4,136** | **.004** |
|  |  |  |  |  |  |  |  |  |  |  |  |  |  |  |  |  |  |  |  |
|  | **LFR** |  |  |  |  |  |  |  |  |  |  |  |  |  |  |  |  |  |  |
|  | Acq | **6.40** | **1,20** | **.012** | 0.77 | 1,20 | .389 | 0.13 | 1,20 | .725 | **27.07** | **10,350** | **<.001** | 1.33 | 10,350 | .214 | 0.84 | 10,350 | .595 |
|  | Trans I | 0.05 | 1,20 | .824 | 0.12 | 1,20 | .730 | 0.07 | 1,20 | .791 | **325.04** | **1,35** | **<.001** | 0.78 | 1,35 | .384 | *3.67* | *1,35* | *.064* |
|  | Rev I | 0.23 | 1,20 | .636 | 0.46 | 1,20 | .504 | 0.20 | 1,20 | .660 | **120.27** | **5,175** | **<.001** | 0.49 | 5,175 | .781 | 1.31 | 5,175 | .262 |
|  | Trans II | 0.46 | 1,19 | .507 | 0.99 | 1,19 | .332 | 0.00 | 1,19 | .977 | **203.14** | **1,34** | **<.001** | 0.57 | 1,34 | .455 | 0.01 | 1,34 | .918 |
|  | Rev II | 0.63 | 1,19 | .438 | 0.71 | 1,19 | .410 | 0.16 | 1,19 | .696 | **71.90** | **4,136** | **<.001** | 0.05 | 4,136 | .996 | 0.22 | 4,136 | .928 |
|  |  |  |  |  |  |  |  |  |  |  |  |  |  |  |  |  |  |  |  |
|  | **TV** |  |  |  |  |  |  |  |  |  |  |  |  |  |  |  |  |  |  |
|  | Hab* | 0.00 | 1,20 | .968 | 0.01 | 1,20 | .909 | 0.11 | 1,20 | .747 | 0.88 | 3,105 | .453 | 0.83 | 3,105 | .480 | 1.91 | 3,105 | .132 |
|  | Acq | *3.36* | *1,20* | *.082* | 0.18 | 1,20 | .675 | 0.00 | 1,20 | .997 | **116.56** | **10,350** | **<.001** | 1.09 | 10,350 | .372 | *1.75* | *10,350* | *.069* |
|  | Trans I | 2.03 | 1,20 | .170 | 0.49 | 1,20 | .494 | 0.77 | 1,20 | .390 | **247.69** | **1,35** | **<.001** | 1.13 | 1,35 | .295 | 0.20 | 1,35 | .654 |
|  | Rev I | 1.21 | 1,20 | .284 | 0.18 | 1,20 | .678 | 0.37 | 1,20 | .551 | **112.93** | **5,175** | **<.001** | 0.94 | 5,175 | .457 | 0.30 | 5,175 | .911 |
|  | Trans II | 0.82 | 1,19 | .378 | 1.53 | 1,19 | .231 | *4.31* | *1,19* | *.052* | **449.54** | **1,34** | **<.001** | 0.95 | 1,34 | .338 | 1.86 | 1,34 | .182 |
|  | Rev II | 0.00 | 1,19 | .947 | 1.36 | 1,19 | .258 | 0.04 | 1,19 | .853 | **212.76** | **4,136** | **<.001** | 1.11 | 4,136 | .353 | 1.80 | 4,136 | .132 |
|  |  |  |  |  |  |  |  |  |  |  |  |  |  |  |  |  |  |  |  |
|  | **LOC** |  |  |  |  |  |  |  |  |  |  |  |  |  |  |  |  |  |  |
|  | Hab* | 2.20 | 1,20 | .154 | 0.00 | 1,20 | .965 | 0.83 | 1,20 | .373 | *2.50* | *3,105* | *.063* | 1.53 | 3,105 | .211 | 0.46 | 3,105 | .711 |
|  | Acq | *3.57* | *1,20* | *.073* | 0.13 | 1,20 | .719 | 0.83 | 1,20 | .373 | **193.31** | **10,350** | **<.001** | 1.23 | 10,350 | .271 | *1.66* | *10,350* | *.089* |
|  | Trans I | 2.11 | 1,20 | .162 | 0.36 | 1,20 | .558 | 0.92 | 1,20 | .350 | **240.42** | **1,35** | **<.001** | 1.14 | 1,35 | .293 | 0.22 | 1,35 | .638 |
|  | Rev I | 1.22 | 1,20 | .283 | 0.08 | 1,20 | .785 | 0.43 | 1,20 | .520 | **69.99** | **5,175** | **<.001** | 0.67 | 5,175 | .650 | 0.42 | 5,175 | .837 |
|  | Trans II | 0.01 | 1,19 | .916 | 2.89 | 1,19 | .106 | 1.45 | 1,19 | .244 | **1075.92** | **1,34** | **<.001** | 0.24 | 1,34 | .629 | *3.64* | *1,34* | *.065* |
|  | Rev II | 0.66 | 1,19 | .426 | 0.07 | 1,19 | .793 | 0.37 | 1,19 | .550 | **255.37** | **4,136** | **<.001** | 0.78 | 4,136 | .539 | **2.84** | **4,136** | **.027** |
|  |  |  |  |  |  |  |  |  |  |  |  |  |  |  |  |  |  |  |  |
|  | **REW** |  |  |  |  |  |  |  |  |  |  |  |  |  |  |  |  |  |  |
|  | Acq | *3.23* | *1,20* | *.087* | 0.20 | 1,20 | .657 | 0.05 | 1,20 | .829 | **3.81** | **10,350** | **<.001** | **2.13** | **10,350** | **.022** | 0.09 | 10,350 | <.999 |
|  | Trans I | 0.35 | 1,20 | .563 | 0.06 | 1,20 | .806 | 0.05 | 1,20 | .825 | **35.37** | **1,35** | **<.001** | 0.32 | 1,35 | .574 | 0.10 | 1,35 | .757 |
|  | Rev I | 0.39 | 1,20 | .539 | 0.00 | 1,20 | .982 | 0.09 | 1,20 | .770 | **35.43** | **5,175** | **<.001** | 0.25 | 5,175 | .941 | 0.92 | 5,175 | .472 |
|  | Trans II | 0.49 | 1,19 | .493 | 0.59 | 1,19 | .452 | 0.88 | 1,19 | .360 | **26.26** | **1,34** | **<.001** | 0.35 | 1,34 | .557 | 1.33 | 1,34 | .258 |
|  | Rev II | 0.20 | 1,19 | .661 | 0.22 | 1,19 | .646 | 1.28 | 1,19 | .272 | **21.52** | **4,136** | **<.001** | 0.77 | 4,136 | .548 | 1.69 | 4,136 | .156 |

*For habituation phase, 4 separate trials were analyzed instead of trial blocks.

Abbreviations: TD, trial duration; LFV, latency to first visit; IVI, inter-visit interval; LFR, latency to first reward; TV, total number of visits; LOC, total number of locations visited; REW, number of rewards found.
